# Improved Siderotic Nodule Detection in Cirrhosis with Susceptibility-Weighted Magnetic Resonance Imaging: A Prospective Study

**DOI:** 10.1371/journal.pone.0036454

**Published:** 2012-05-10

**Authors:** Wei Chen, Zachary DelProposto, Dongmei Wu, Jian Wang, Quan Jiang, Stephanie Xuan, YongQuan Ye, Zishu Zhang, Jiani Hu

**Affiliations:** 1 Department of Radiology, Southwest Hospital, Third Military Medical University, Chongqing, China; 2 Department of Radiology, Henry Ford Hospital, Detroit, Michigan, United States of America; 3 Shanghai Key Laboratory of MR, Physics Department, East China Normal University, Shanghai, China; 4 Department of Neurology, Henry Ford Hospital, Detroit, Michigan, United States of America; 5 Faculty of Arts & Science, University of Toronto, Toronto, Ontario, Canada; 6 Department of Radiology, Wayne State University, Detroit, Michigan, United States of America; Mayo Clinic College of Medicine, United States of America

## Abstract

**Background:**

Hepatic cirrhosis is a common pathway of progressive liver destruction from multiple causes. Iron uptake can occur within the hepatic parenchyma or within the various nodules that form in a cirrhotic liver, termed siderotic nodules. Siderotic nodule formation has been shown to correlate with inflammatory activity, and while the relationship between siderotic nodule formation and malignancy remains unclear, iron distribution within hepatic nodules has known implications for the detection of hepatocellular carcinoma. We aimed to evaluate the role of abdominal susceptibility-weighted imaging in the detection of siderotic nodules in cirrhotic patients.

**Methodology/Principal Findings:**

Forty-six (46) cirrhotic patients with at least one siderotic nodule detected on previous imaging underwent both computed tomography and magnetic resonance imaging (T1-, T2-, T2*-, and susceptibility-weighted imaging) at 3.0 Tesla. Imaging data was independently analyzed by two radiologists. Siderotic nodule count was determined for each modality and imaging sequence. For each magnetic resonance imaging technique, siderotic nodule conspicuity was assessed on a 3 point scale (1 = weak, 2 = moderate, 3 = strong). More nodules were detected by susceptibility weighted imaging (n = 2935) than any other technique, and significantly more than by T2* weighted imaging (n = 1696, p<0.0001). Lesion conspicuity was also highest with susceptibility-weighted imaging, with all nodules found to be moderate (n = 6) or strong (n = 40); a statistically significant difference (p<0.001).

**Conclusions:**

Susceptibility-weighted imaging had the greatest lesion conspicuity and detected the highest number of siderotic nodules suggesting it is the most sensitive imaging technique to detect siderotic nodules in cirrhotic patients.

## Introduction

Iron is a critical element for human life, and the majority of human iron exists in heme-based proteins (predominantly hemoglobin) or ferritin, an iron storage protein. The liver is the primary organ for iron storage and metabolism. Numerous human diseases are associated with abnormalities of iron metabolism, and in the liver, the pathogenesis of many hepatic diseases result in the accumulation of excess hepatic iron. Excess hepatic iron can result from genetic predisposition (hemochromatosis) or from excess circulating iron, which is typically due to excess breakdown of hemoglobin (hemosiderosis). Many techniques have been developed to detect and quantify hepatic iron content because of its importance in the diagnosis and prognosis of related diseases and for monitoring and evaluating treatment response. The quantification of iron content is most commonly indirectly estimated by serum ferritin level and directly determined by liver biopsy. However, ferritin measurements often do not accurately reflect hepatic iron stores [1], [2]. Liver biopsy is invasive, entails risks with repeated use, samples only a small portion of the liver, and is not an option in all patients [3]–[5]. Biopsy is also problematic, as iron distribution is often inhomogeneous, particularly in diseased livers. As a result of these issues, and with improvements in imaging technology, T2* mapping with MRI has become a viable method for estimating iron concentration within the liver [6].

Hepatic nodules can be detected with ultrasound, computed tomography (CT), or magnetic resonance (MR) imaging. Ultrasound lacks the ability to discriminate iron content, and the sensitivity of computed tomography for the detection of iron is also limited [7]. MR T2* weighted imaging, on the other hand, is considered to be the most sensitive technique for the visualization of iron-containing hepatic nodules, while T2* mapping methods can be used to quantify diffuse hepatic iron concentration. Susceptibility-weighted imaging (SWI) is more sensitive to detecting focal iron rich lesions by adding phase information in addition to T2* contrast. It has been well documented in the brain that SWI is superior to T2* and other existing MRI techniques for the detection of iron content, hemorrhage, and calcification [8], [9]. This is because phase data is an additional source of information about local susceptibility changes induced by iron, calcium or deoxyhemoglobin in various physiological or pathological conditions. For example, SWI can detect as many as 5 times more cerebral hemorrhagic lesions in patients with diffuse axonal injury than conventional T2* weighted imaging [10].

Hepatic cirrhosis is the progressive destruction of normal hepatic architecture with fibrosis and replacement by a range of partially restorative nodules, varying from benign regenerative nodules and premalignant dysplastic nodules to hepatocellular carcinoma. Even in the absence of systemic iron storage diseases such as hemochromatosis, iron can accumulate within regenerative or dysplastic nodules in the cirrhotic liver; these nodules are termed “siderotic nodules” (SN) [11]. While the mechanism of iron deposition (siderosis) within reticuloendothelial cells by mobilized iron from damaged hepatocytes in systemic iron storage diseases has been characterized, the process of siderotic nodule formation remains uncertain though both active viral replication and transferrin receptor abnormalities are thought to play a role [12]–[14]. Though only a small percentage of siderotic nodules are dysplastic, approximately 25% of all dysplastic nodules (DN) are also siderotic nodules [15], [16]. Moreover, dysplastic SN are pre-malignant lesion while regenerative SN are markers for severe viral or alcoholic cirrhosis. Therefore, the diagnosis of siderosis is clinical important. The relationship of hepatic iron deposition to hepatic fibrosis, cirrhosis and neoplasia is also not fully understood [17], [18]. Magnetic resonance imaging is currently unable to differentiate siderotic regenerative nodules from siderotic dysplastic nodules [19]. The association of siderotic nodules and malignancy remains controversial; some studies reported no increase in dysplastic nodules or hepatocellular carcinoma in patients with siderotic nodules [15], [20] but others have reported that siderotic nodules can be precursors of primary hepatocellular carcinomas in patients with chronic liver diseases [21]–[23], and that the incidence of hepatocellular carcinomas is higher in patients with iron-containing nodules than in those without iron-containing nodules [23]. Iron-free foci were found in siderotic macroregenerative nodules in liver cirrhosis, and these foci were classic and overt carcinoma or borderline lesions showing an expansive growth pattern [15]. Furthermore, the displacement of iron within a nodule by tumor is an established method for the early detection of HCC [16], [21], [24]. Therefore, the diagnosis of siderosis, siderotic nodules, and iron-free nodules in the cirrhotic liver are clinically relevant for the detection of hepatocellular carcinoma.

T2*-weighted imaging is currently the most sensitive MRI technique to detect siderotic nodules in the liver with a reported 80% sensitivity and 95% specificity [19]. A sensitive and noninvasive technique could also help answer questions regarding the iron involvement in the development of hepatocellular carcinoma (HCC) by monitoring iron content in multistep human HCC: from low-grade dysplastic nodule, to high-grade dysplastic nodule, early HCC, well-differentiated HCC, nodule-to-nodule HCC, and finally, to moderately differentiated HCC [25], [26]. Technical limitations have previously precluded the use of SWI in the abdomen; however, with the recent availability of a multislice 2D breath-hold technique, abdominal SWI has become feasible [27]. The goal of this study is to evaluate whether abdominal SWI allows greater sensitivity for the detection of siderotic nodules than other CT and MRI techniques, including T2* weighted gradient-echo MR imaging.

## Materials and Methods

### Ethics Statement

All research procedures were approved by the Institutional Review Board of the Third Medical Military University and were conducted in accordance with the Declaration of Helsinki. Written informed consent was obtained for all patients.

### Participants

Using an institutional database, a total of 362 patients from December 2009 to December 2010 with clinically diagnosed cirrhosis were identified. From this set of identified patients, 46 patients (29 men, 17 women, mean age: 47 years, range: 28–76 years) agreed to participate in our study and have a specialized set of CT and MR examinations. Patient inclusion was contingent upon meeting the following criteria: 1) ability to sustain a 20 second breath hold; 2) no evidence of iron overload (e.g., hemochromatosis or hemosiderosis); 3) at least one siderotic nodule previously detected by CT or MR; and 4) no other lesions, such as cholangiocarcinoma, detected by prior MR or CT; however, patients with hepatocellular carcinoma were included in the study. Of the 46 patients, causes of cirrhosis were hepatitis B (n = 37, 80.4%), hepatitis C (n = 5, 10.9%), autoimmune cirrhosis (n = 2, 4.3%), alcohol abuse (n = 1, 2.2%), and primary biliary cirrhosis (n = 1, 2.2%).

### Computed Tomographic Imaging

All subjects were imaged with dual-source CT (Definition, Siemens Healthcare, Forchheim, Germany). Imaging parameters were: 250 mAs, 120 kVp, 1.2 mm beam collimation with a 0.5 s gantry rotation time. Field of view (FOV) was 35 cm, with a reconstruction thickness and interval both of 5 mm. No oral contrast was administered. The examination consisted of noncontrast images followed by three dynamic images acquired 35 s (hepatic arterial phase), 70 s (portal venous phase), and 180 s (delayed phase) acquired following the intravenous administration of 100–120 ml Ultravist 370 (Bayer-Schering, Leverkusen, Germany) at a rate of 3–4 ml/s.

**Figure 1:**
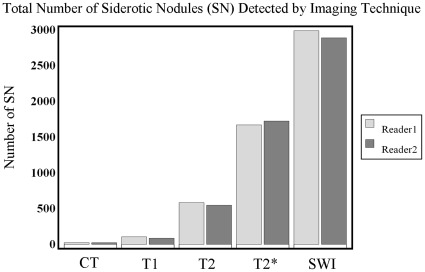
Total number of siderotic nodules (SN) detected by imaging technique for both readers. The total number of siderotic nodules detected by imaging for all patients by two independent readers. Different imaging methods are represented on the horizontal axis. The number of detected siderotic nodules is represented on the vertical axis. Light gray: results for reader 1. Dark gray: results for reader 2.

**Table 1:** Number of siderotic nodules (SN) detected by all tested imaging techniques.

Parameter	CT	T1-weighted imaging	T2-weighted imaging	T2*-weighted imaging	SWI§
Mean Count†	0.5±0.2	2.1±1.7	12.2±6.6	36.9±15.4	63.8±19.7
*P*-value‡	<0.0001	<0.0001	<0.0001	<0.0001	-

† The mean value for both readers are listed ± standard errors of the mean for all patients.

‡ P-values were calculated using paired t-tests between SWI and each other imaging technique.

§ SWI: susceptibility-weighted imaging, a 2D-gradient echo based abdominal imaging technique.

### Magnetic Resonance Imaging

MR imaging for all subjects was performed on a 3.0 T whole-body system (Magnetom Trio, Siemens Healthcare, Erlangen, Germany) using a standard 12-channel matrix coil, without intravenous contrast enhancement. The following MR pulse sequences were used for all patients: transverse T1-weighted 2D gradient echo (GRE) (flip angle 70°, TR/TE 140/2.46 ms), transverse T2-weighted 2D fast spin echo (flip angle 122°, TR/TE = 3700/84 ms, ETL 9), transverse T2*-weighted 2D GRE (flip angle 20°, TR/TE = 150/10 ms) and transverse abdominal 2D SWI (flip angle 20°, TR/TE = 150/10 ms). For all sequences, FOV was 280×285 mm^2^ and matrix was 320–384 × 250; slice thickness was 5 mm with a gap of 1 mm. Depending upon the liver size, two to three breath holds of 15 to 20 s duration were used for each sequence.

For abdominal SWI, a recently developed multislice 2D GRE sequence with SWI reconstruction (Siemens work-in-progress sequence #608, Siemens Healthcare, Erlangen, Germany) was used. An SWI dataset consisted of 3 contiguous 10-slice transverse acquisitions through the liver, with the duration of each acquisition suitable for a single breath-hold (less than 20 s).

SWI postprocessing was performed automatically on the magnet. Postprocessing of abdominal SWI images was designed to reduce motion artifacts from breathing and cusp artifact (also termed singularity artifact) from B_0_ inhomogeneity, both of which are increased in abdominal imaging compared to brain imaging. First, complex images from each of the 12 channels were acquired individually and high-pass filtered with a 32×32 filter to reduce artifacts related to both coil sensitivity and magnetic field inhomogeneity. High-pass filtering was achieved by the following steps: 1) Fourier transformation of the complex image to k-space; 2) while keeping the center of k-space (size 32×32) unchanged, the remainder of the k-space matrix was zero-filled; 3) inverse Fourier transform of the zero-filled k-space to obtain a complex low-pass filter, and division of the original complex image with this low-pass filter to remove low spatial frequency components. Then, the high-pass filtered complex images from each channel were weighted by the coil sensitivity factor and combined to generate a single complex image, as described by the adaptive combine method [28]. The phase image from this final channel-combined complex image was extracted and used to create a positive phase mask such that the mask value would be 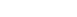 for phase >0 and be 1 otherwise. Finally, the SWI image was created by multiplying this phase mask four times to the magnitude image [29]. The main difference between this process and brain SWI [29] is the filtering of the multichannel data prior to recombination. Additionally, SWI parameters such as resolution, flip angle (the main determinant of contrast in magnitude images), and echo time (main determinate of contrast for phase images) were evaluated to determine the optimal experimental parameters for hepatic imaging in a typical clinical setting.

**Figure 2:**
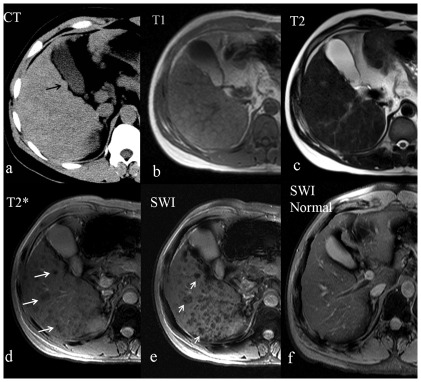
Representative images from a single patient comparing CT and multiple MR imaging methods. A 37 year-old man with hepatitis-B induced cirrhosis. (a) CT image demonstrates a high-attenuation siderotic nodule in the gallbladder fossa (black arrow). (b) Transverse T1-weighted gradient echo image and (c) Transverse T2-weighted fast spin echo image with a few hypointense nodules. (d) Transverse T2*-weighted GRE image demonstrated numerous hypointense nodules with different sizes (long arrows). (e) Transverse susceptibility weighted image (SWI) shows a greater number of hypointense lesions, with a higher contrast relative to background parenchyma (short arrows). (f) SWI comparison image from a normal 37-year-old man.

**Table 2:** Siderotic Nodule (SN) Distribution.

NoduleDistribution	CT	T1-weighted imaging	T2-weighted imaging	T2*-weighted imaging	SWI†
focal	11.5±0.7	17.5±0.7	29.5±2.1	18.5±2.1	0
scattered	0	3±2.8	6.5±2.2	21.5±3.5	10.5±2.1
diffuse	0	0	0	6±1.4	35.5±2.1

Nodule distribution was classified as focal (<5 per slice), scattered (5–20 per slice), or diffuse (>20 per slice). The average number of patients and its standard deviation in each level determined by two readers are listed.

† SWI: susceptibility-weighted imaging, a 2D-gradient echo based abdominal imaging technique.

### Image Analysis

Nodules that were hyperattenuating compared to background parenchyma were considered to represent siderotic nodules on CT, and nodules that were hypointense to background parenchyma were considered to represent SN on MRI [22], [30]. Because of the large numbers and small sizes of these nodules, it was not feasible to evaluate all images and all imaging sequences by enumerating SN in the entire liver. By reader consensus, a single representative slice was first chosen from the T2*-weighted GRE dataset that contained the largest number of SN; then, corresponding slices which matched or most closely matched the representative slice were chosen from the T1-weighted, T2-weighted, SWI, and CT datasets by comparing the position of the liver.

After the combined representative dataset was created for each patient, two readers independently reviewed all 92 (46 MRI, 46 CT) examinations on a commercially available imaging workstation (Syngo, Siemens Medical Solutions). Readers were blinded to previous clinical MR interpretations, pathologic results, and sequences used. For each of the representative slices, the number of SN was determined and the distribution classified as focal (<5 SN per slice), scattered (5–20 SN per slice), or diffuse (>20 SN per slice). For any questionable nodule, consensus review with a third reader was used to decide. Any nodule detected on CT with an attenuation value ≥100 Hounsfield units was considered to represent a calcified lesion and was excluded for SN analysis [31]. For MR images, the conspicuity of SN was calculated based on the ratio of SN signal intensity to background parenchyma and graded on a 1 to 3 scale: grade 1 (low conspicuity), >0.7; grade 2 (moderate conspicuity), conspicuity ratios 0.4 and 0.7; grade 3 (high conspicuity), conspicuity ratio <0.4.

### Statistical Analysis

All statistics were computed using dedicated statistical software (SPSS version 13.0, SPSS Inc., Chicago, Illinois). P values of <0.05 were considered statistically significant. The number, distribution, and conspicuity of SN were compared by paired *t*-test. Cohen’s kappa method was used to assess inter-reader agreement with respect to diagnosis determined by each reader on the basis of images of different imaging techniques. The reported Cohen Κ is the average Κ values computed for both readers over the entire set of all images.

## Results

The comparison of SN detected by CT, T1-, T2-, T2*-weighted and SWI imaging is illustrated in Figure 1, for both independent readers. Using reader averages, the number of SN per patient ranged from 0 to 3.5 for CT images, 0 to 13.5 for T1-weighted images, 0 to 80.5 for T2-weighted images, 5 to 199 for T2*-weighted images, and 8 to 305 for SWI. The average number of SN detected for all patients was significantly greater on SWI images than on any other imaging technique (p<0.0001); refer to Table 1. An example showing the number and conspicuity of nodules seen with T2* and SWI compared to other techniques is shown in Figure 2. There was no single case where SWI detected fewer SN than any other imaging technique. In this study, SN were detected by T2*-weighted and SWI imaging in each patient. Using averaged reader results, however, SN could not be detected by CT in 75% (34.5/46) of patients, by T1-weighted imaging in 55% (25.5/46) of patients, and T2-weighted imaging in 22% (10/46) of patients (Table 2). Nodule distribution varied by technique. According to the nodule distribution classification, more patients were classified as diffuse 77% (35.5/46) by SWI, while T2* GRE demonstrated a diffuse distribution of nodules in 13% (6/46) of patients (Table 2).

**Table 3:** Siderotic Nodule (SN) Conspicuity Grading.

Score	T1-weighted imaging	T2-weighted imaging	T2*-weighted imaging	SWI†
1 (weak)	17.5±0.7	29.5±2.1	6.5±0.7	0
2 (moderate)	3±2.8	5±1.4	27.5±0.7	6±0.7
3 (strong)	0	1.5±0.7	12±0	40±1.4

The number of patients (mean for two readers ± standard deviation) is listed.

† SWI: susceptibility-weighted imaging, a 2D-gradient echo based abdominal imaging technique.

**Figure 3:**
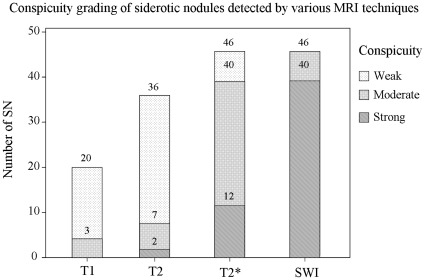
Conspicuity grading of siderotic nodules by various magnetic resonance imaging techniques. Results indicate the mean number (vertical axis, for both readers) of siderotic nodules (SN) and the relative conspicuity grade.

Overall conspicuity of SN was categorized for each MR imaging technique (Table 3). Lesion conspicuity was lowest with T1-weighted imaging and greatest with SWI. Conspicuity was found to be significantly different between every pair of techniques (p<0.001). Compared to T2*-weighted imaging, SN detected with SWI had superior contrast (lesions appeared more hypointense to background hepatic parenchyma) and visibility (Fig. 3). Nearly all (87%, 40/46) patients were given a conspicuity grade of 3 (the highest) on SWI, compared to 26% (12/46) for T2*-weighted imaging. Similarly, no patients were considered to have grade 1 conspicuity on SWI imaging, whereas 14% (6.5/46) were considered grade 1 on T2*-weighted images. Because conspicuity on CT has a different definition than that from MR images, conspicuity of SN on CT images was not included in the comparison.

Inter-reader correlation for conspicuity, determined using the Κ coefficient, was estimated at 0.82 based on results provided by each individual reader.

## Discussion

Our results of this study evaluating 46 patients with hepatic cirrhosis and siderotic nodules demonstrated that abdominal SWI detects more nodules than other tested sequences; in particular, almost six times as many patients (35.5 vs. 6, using averaged reader statistics, Table 2) were categorized as having diffuse SN (>20 nodules per slice) than T2*-weighted images with long echo times (TE), the current standard. Not only was SWI able to detect more lesions, but SWI also demonstrated significantly better lesion conspicuity than other tested sequences. These findings suggest that SWI has a greater sensitivity for the detection of SN than T2*-weighted imaging, likely due to the improved sensitivity for the detection of iron content. At 3T, both SWI and T2*-weighted images have an echo time of 10 ms; which is equivalent to an echo time of 20 ms at 1.5T when considering susceptibility effects. T2*-weighted demonstrates 80% sensitivity for SN detection at 1.5 T, and longer echo times (e.g., 15 ms at 1.5T) may further increase sensitivity [17], [22]. Increased conspicuity on SWI images can be explained by the enhancement of susceptibility effects when using processed phase information in combination with magnitude images. Previous theoretical work has indicated that even an object measuring less than 25% of a voxel can have a conspicuous appearance if sufficiently paramagnetic [32].

Improved detection of siderotic nodules has possible clinical uses and opens up new potential avenues for research, such as evaluating disease activity and progression as well as malignancy. Hepatic iron content is a known risk factor for developing hepatocellular carcinoma (HCC). While the association between grades of siderotic dysplastic nodules and malignancy has not been established, siderotic dysplastic nodules are thought to represent premalignant lesions [15], [16], [20], [21]. MRI has been used to grade siderotic nodules, and nodule grade and number have been shown to be significantly correlated with periportal inflammatory activity in cirrhotic patients [33]. The distribution of iron and of siderotic nodules within the liver can enhance HCC detection; regions of hepatic parenchyma with a relative paucity of iron or siderotic nodules sparing are more likely to contain HCC [34]. The pattern of iron distribution within nodule can improve the detection of HCC; decreased iron content within an otherwise siderotic nodule (referred to as a “nodule within a nodule”) is highly likely to represent HCC [16], [24]. Therefore, improving the sensitivity of iron detection and distribution within all types of hepatic nodules could improve the ability to detect hepatocellular carcinoma and disease activity. We speculate that percutaneous hepatic biopsy of some indeterminate hepatic nodules may be obviated if a nodule not definitively classified as siderotic on other imaging techniques could be shown to be diffusely siderotic with more sensitive imaging techniques such as SWI. Additionally, SWI may also be of use to evaluate iron loading within hepatic parenchyma in diseases of iron overload.

We are aware of several limitations in this study. While T2* weighted imaging is the most common clinically used method to detect siderotic nodules, the gold standard for siderotic nodule detection remains histologic; pathological correlation with explant livers would be the ideal reference; lack of pathologic correlation potentially biases results to favor T2* and SWI over other imaging techniques. We also considered all hypointense lesions on T2* and SWI to be siderotic nodules; while this is most likely given our patient subset, other lesions can also appear hypointense. Only histologic explant liver evaluation could definitively exclude all possible nonsiderotic hypointense nodules. The inclusion of patients only with known siderotic nodules introduces a bias, and our evaluation cannot conclusively prove whether SWI would detect siderotic nodules in instances where other MRI imaging methods would detect none; T2* weighted MRI detected at least 5 or more siderotic nodules in all patients. Since only a single representative image from each examination was chosen for evaluation, the potential for undersampling bias exists; evaluation of the entire hepatic volume would have eliminated this possible bias. The use of multiple breath-hold imaging acquisitions to achieve coverage of the entire liver may be difficult or unattainable in some cirrhotic patients, such as those with ascites or reduced pulmonary function. Gastrointestinal air is a known source of magnetic susceptibility and can impart artifacts, and can limit evaluation of some liver regions depending upon anatomic considerations; however, we anticipated that further technological improvements in MR sequence design can minimize such artifacts.

In conclusion, we show in this study that imaging using SWI substantially improves both the conspicuity of siderotic nodules and number of detected nodules relative to other techniques, including T2*-weighted gradient-echo imaging; additionally, these results are repeatable as inter-reader agreement is highly correlated. SWI should be a useful addition to current imaging techniques, and may prove to be the most sensitive technique for the detection and characterization of hepatic siderotic nodules in the future.
